# Distinguishing Cerebral Infarction With Neck Pain and Hemiparesis From Cervical Spinal Epidural Hematoma Without MRI: A Case Report

**DOI:** 10.7759/cureus.61931

**Published:** 2024-06-08

**Authors:** Tatsuya Tanaka, Junpei Kato, Tomoyuki Naito, Tomihiro Wakamiya, Kimihiro Nakahara, Takashi Agari, Yuhei Michiwaki, Takashi Sugawara, Hiroshi Itokawa, Kazuaki Shimoji, Eiichi Suehiro, Keisuke Onoda, Akira Matsuno, Tadatsugu Morimoto

**Affiliations:** 1 Neurosurgery, International University of Health and Welfare, Narita Hospital, Narita, JPN; 2 Neurosurgery, International University of Health and Welfare Narita Hospital, Narita, JPN; 3 Orthopedic Surgery, Faculty of Medicine, Saga University, Saga City, JPN

**Keywords:** stroke mimic, ct myelography, cervical spinal epidural hematoma, neck pain, misdiagnosis

## Abstract

In patients presenting neck pain and hemiparesis, differentiation between cerebral infarction and cervical spinal epidural hematoma is vital yet challenging, particularly when magnetic resonance imaging (MRI) is not feasible. A 59-year-old woman presented with a sudden onset of left-sided hemiparesis and neck pain. MRI was contraindicated because the patient underwent embolization in childhood. Head computed tomography (CT) revealed no evidence of hemorrhage or early ischemic signs. Cervical CT revealed no evidence of hematoma within the spinal canal. Myelography and CT myelography revealed no significant cervical spine abnormalities. The diagnosis was cerebral infarction. Cervical spine MRI is the gold standard examination for diagnosing cervical spinal epidural hematoma, but cervical spine CT, myelography, and CT myelography may be useful when MRI is contraindicated.

## Introduction

Stroke is a common neurological disorder characterized by the sudden onset of neurological deficits due to occlusion or rupture of cerebral arteries. Thrombolytic therapy with alteplase within 4.5 hours of ischemic stroke onset and mechanical thrombectomy for large vessel occlusion has been proven effective in improving patients' clinical outcomes [1,2]. It is important to distinguish the ischemic stroke from stroke mimics because the treatable time window is narrow.

Stroke mimics are a group of diseases that exhibit stroke-like clinical features, such as acute onset and localized neurological deficits due to symptoms caused by diseases other than cerebrovascular disorders [3,4]. Cervical epidural hematoma can mimic stroke, and intravenous thrombolysis can have disastrous consequences [3-10].

In this report, we present a case of neck pain, hemiparesis, and lack of cranial nerve symptoms in which differentiating between cerebral infarction and cervical spinal epidural hematoma was difficult because of magnetic resonance imaging (MRI).

## Case presentation

**Figure 1:**
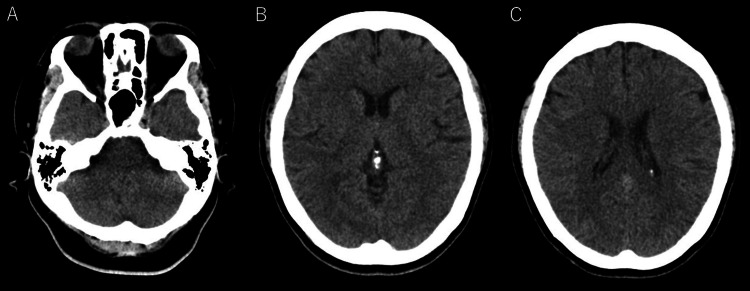
(A-C) Initial head computed tomography showing no evidence of hemorrhage and early ischemic signs.

A 59-year-old woman presented with a sudden onset of left-sided hemiparesis and neck pain without any triggering factors. The patient had a history of patent ductus arteriosus, hypertension, and neck pain and no history of stroke or bleeding tendency. She was taking nifedipine, telmisartan, metoprolol tartrate, and etoricoxib. MRI was contraindicated because the patient underwent embolization in China during childhood. The patient was brought to the Emergency Department 4.5 hours after the onset of symptoms. Vital signs on arrival were as follows: blood pressure, 212/108 mmHg; temperature, 36.3 °C; pulse, 73 beats/minute; respiration rate, 18 breaths/minute; and oxygen saturation, 99%. Physical examination revealed neck stiffness and left-sided hemiparesis without any cranial nerve abnormalities. Neurological examination revealed a Glasgow Coma Scale score of 15, intact sensation throughout the face and bilateral extremities, no effort in the left arm and leg against gravity, and muscle strength of 5/5 on manual muscle testing for the right arm and leg. Tendon reflex and Babinski sign test results were normal. The National Institutes of Health Stroke Scale (NIHSS) score was 6. The initial laboratory examination revealed a blood sugar level of 136 mg/dL, and coagulation functions such as prothrombin time, activated partial thromboplastin time, D-dimer, and platelet count were normal. The electrocardiogram was normal, and no atrial fibrillation was observed. Head computed tomography (CT) revealed no evidence of hemorrhage or early ischemic signs (Figure 1).

**Figure 2:**
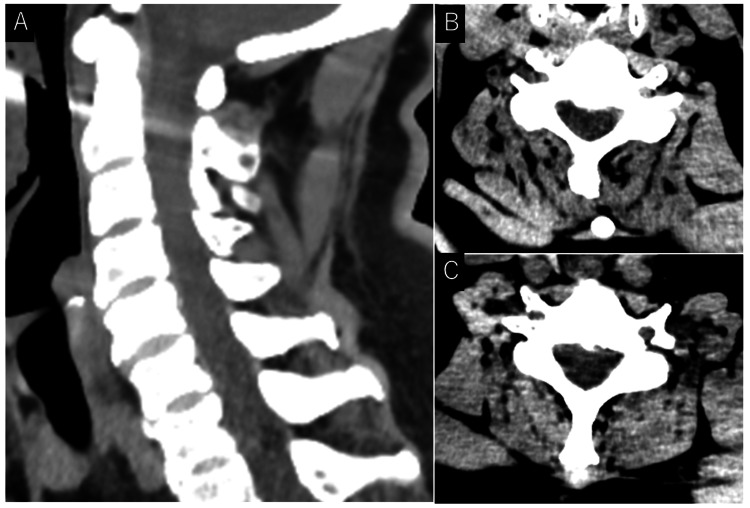
Cervical computed tomography. (A) Sagittal view and (B: C5 level and C: C6 level) axial views of cervical computed tomography showing no hematoma within the spinal canal.

Cervical CT revealed no evidence of hematoma within the spinal canal (Figure 2).

**Figure 3:**
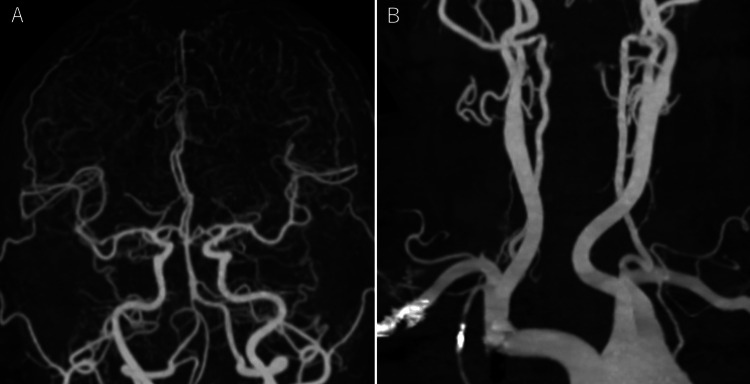
Three-dimensional computed tomography angiography showing no stenosis or dissection of the (A) cerebral or (B) carotid arteries.

CT angiography revealed no stenosis or dissection of the carotid or cerebral arteries (Figure 3).

**Figure 4:**
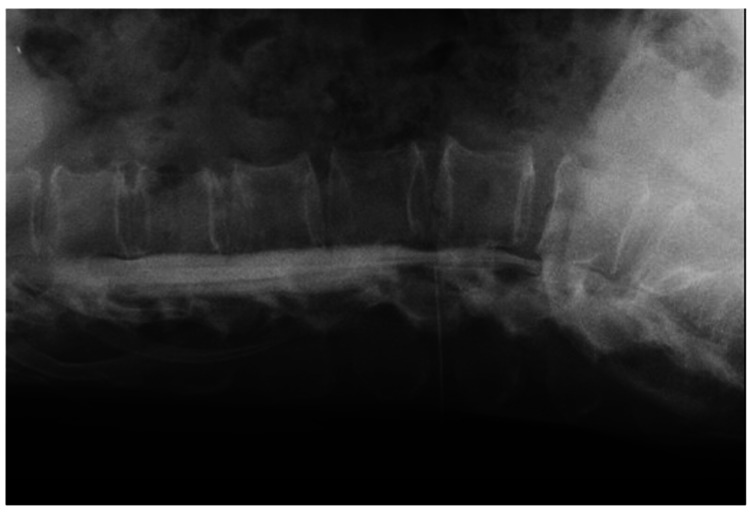
Myelography: Lateral view myelography image showing a 23-G needle inserted between the L3/4 spinous processes under fluoroscopy and 10 mL of contrast injected.

Therefore, myelography and CT myelography were performed to differentiate spinal cord symptoms caused by cervical spinal epidural hematoma or cervical disk herniation from those caused by cerebral infarction. A 23-gage needle was inserted between the L3/4 spinous processes under fluoroscopy, 10 mL of cerebrospinal fluid was collected, and 10 mL of contrast dye was injected (Figure 4).

**Figure 5:**
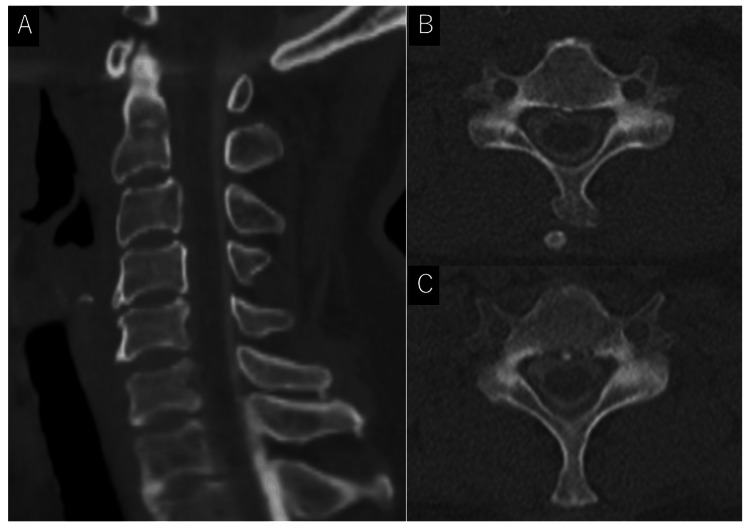
Cervical computed tomography myelography. (A) Sagittal view and (B: C5 level and C: C6 level) axial views of cervical CT myelography showing no hematoma and no lesions compressing the dural canal within the spinal canal.

CT myelography revealed no significant cervical spine abnormalities (Figure 5).

The most likely diagnosis was ischemic stroke, particularly lacunar infarction.

Antiplatelet and brain protection therapies were administered. Aspirin (100 mg/day) was orally administered, and ozagrel Na (160 mg/day) and edaravone (60 mg/day) were intravenously administered.

After two days, the patient fully recovered, the neck pain resolved, and the NIHSS score was 0. The patient was discharged seven days later.

## Discussion

Differentiation between cervical spinal epidural hematoma with hemiparesis and acute stroke

It is crucial to include a cervical spinal epidural hematoma in the differential diagnosis of patients experiencing sudden neck pain and subsequent hemiparesis [6,9,10]. Misdiagnosing a cervical spinal epidural hematoma as a stroke can result in poor clinical outcomes due to inappropriate treatment through antithrombotic therapy [3-10].

Spontaneous cervical epidural hematoma is a rare disease, with an estimated annual incidence of 0.1 in 100,000 [11]. Typical symptoms of cervical spinal epidural hematoma are quadriplegia and paraplegia. However, the hematoma may compress only the ipsilateral asymmetrical spinal cord, and it has been reported that 2.8% to 6.9% of patients present with Brown-Séquard syndrome or hemiparesis mimicking acute stroke [12,13].

The onset of neck or back pain and the absence of cranial nerve signs have been suggested as important indicators for diagnosing cervical spinal epidural hematoma with hemiparesis [6,9,10]. However, a recent review found that 16 out of 51 cases presented with hemiplegia without neck pain, and 6 out of 51 cases presented with hemiplegia and dysarthria [8].

Differentiating between cervical spinal epidural hematoma with hemiparesis and acute stroke is crucial, not only based on symptoms and medical history but also through imaging.

Imaging diagnosis of cervical spinal epidural hematoma with hemiparesis

Plain CT of the cervical spine is a valuable tool in diagnosing spinal epidural hematoma [8,10,11,14-16]. CT findings of this hematoma vary depending on its phase. In the acute phase, the lesion is dense compared to the spinal cord. In soft tissue imaging, CT images enhance tissue and hematoma differentiation. Air or metal artifacts increase the risk of misdiagnosis. Although plain CT also provides acceptable results, the potential for misdiagnosis remains with this modality [8,10,11,14-16].

MRI is considered the most reliable diagnostic tool for diagnosing spinal epidural hematoma [6,8,15]. During the first 24 hours, these hematomas are isointense on T1-weighted (T1WI) and mildly hyperintense on T2-weighted (T2WI) imaging. After 72 hours, due to hemoglobin oxygenation status and cell lysis, high and low signals appear at T1WI and T2WI, respectively [15]. MRI is a non-invasive imaging examination that lacks ionizing radiation and high contrast resolution compared to CT. Additionally, it has been reported that cervical MR angiography source images are useful for rapid screening of spinal epidural hematoma [17]. The source image of MR angiography is the time-of-flight close to the T1WI signal, which is considered to correspond to the signal intensity of acute-phase hematoma.

Conventional and CT myelography may be alternatives to MRI in MRI-contraindicated patients. Myelography is a procedure in which a lumbar puncture is performed, and a contrast agent is delivered percutaneously into the subarachnoid space through a spinal needle, thereby enhancing visualization of the contents within the spinal canal. Because it provides images with higher spatial resolution than conventional MR images, CT myelography can depict the subdural and subarachnoid space in detail, including visualization of small nerves [18,19].

Diagnostic procedure for MRI-contraindicated patients with neck pain and hemiparesis

Cervical spinal epidural hematoma and acute ischemic stroke with hemiparesis are difficult to diagnose in patients who cannot undergo MRI imaging, such as in this case.

Typical symptoms of cervical epidural hematoma with hemiparesis are neck pain and absence of cranial nerve symptoms [6,9,10]. These symptoms are similar to hemorrhagic stroke, including subarachnoid hemorrhage, and ischemic stroke, including arterial dissection. First, a head and neck CT scan is performed to check for hemorrhagic stroke. Next, three-dimensional computed tomography angiography (3DCTA) of the head and neck vessels, including the aortic arch, is performed to check for arterial dissection.

If artery stenosis is suggested, the cause may be misdiagnosed as acute ischemic stroke associated with arterial dissection. To avoid this misdiagnosis, it is important to consider whether the symptoms can be explained by arterial narrowing and identify areas of abnormally high density within the spinal canal.

Cervical spinal epidural hematoma without neck pain or absence of cranial nerve symptoms has also been reported, making it extremely difficult to differentiate between ischemic stroke and cervical spinal epidural hematoma [8].

If differential diagnosis is difficult due to artifacts, it is better to perform myelography and CT myelography to check for lesions compressing the dural canal within the spinal canal.

## Conclusions

We reported a case of cerebral infarction presenting with neck pain, hemiparesis, and lack of cranial nerve symptoms that were difficult to differentiate from cervical epidural hematoma because the MRI was contraindicated.

As it is difficult to diagnose based on symptoms alone, early diagnosis with imaging is necessary. MRI is the gold standard examination for the diagnosis of cervical spinal epidural hematoma, but cervical spine CT, myelography, and CT myelography may be useful when MRI is contraindicated.
